# *Pseudomonas aeruginosa* Activates Quorum Sensing, Antioxidant Enzymes and Type VI Secretion in Response to Oxidative Stress to Initiate Biofilm Formation and Wound Chronicity

**DOI:** 10.3390/antiox13060655

**Published:** 2024-05-27

**Authors:** Jane H. Kim, Julianna Dong, Brandon H. Le, Zachery R. Lonergan, Weifeng Gu, Thomas Girke, Wei Zhang, Dianne K. Newman, Manuela Martins-Green

**Affiliations:** 1 Department of Molecular, Cell and Systems Biology, University of California, Riverside, CA 92521, USA; 2 Institute for Integrative Genome Biology, University of California, Riverside, CA 92521, USA; 3 Department of Botany and Plant Sciences, University of California, Riverside, CA 92521, USA; 4 Division of Biology and Biological Engineering, California Institute of Technology, Pasadena, CA 91125, USA; 5 Geological and Planetary Sciences, California Institute of Technology, Pasadena, CA 91125, USA

**Keywords:** anaerobic respiration, bacteria, antioxidant enzymes, RNAseq, chronic wounds

## Abstract

*Pseudomonas aeruginosa* (*PA*) is an opportunistic pathogen frequently isolated from cutaneous chronic wounds. How *PA*, in the presence of oxidative stress (OS), colonizes chronic wounds and forms a biofilm is still unknown. The purpose of this study is to investigate the changes in gene expression seen when PA is challenged with the high levels of OS present in chronic wounds. We used a biofilm-forming *PA* strain isolated from the chronic wounds of our murine model (RPA) and performed a qPCR to obtain gene expression patterns as RPA developed a biofilm in vitro in the presence of high levels of OS, and then compared the findings in vivo, in our mouse model of chronic wounds. We found that the planktonic bacteria under OS conditions overexpressed quorum sensing genes that are important for the bacteria to communicate with each other, antioxidant stress genes important to reduce OS in the microenvironment for survival, biofilm formation genes and virulence genes. Additionally, we performed RNAseq in vivo and identified the activation of novel genes/pathways of the Type VI Secretion System (T6SS) involved in RPA pathogenicity. In conclusion, RPA appears to survive the high OS microenvironment in chronic wounds and colonizes these wounds by turning on virulence, biofilm-forming and survival genes. These findings reveal pathways that may be promising targets for new therapies aimed at disrupting *PA*-containing biofilms immediately after debridement to facilitate the treatment of chronic human wounds.

## 1. Introduction

Cutaneous wound healing involves not only the cells of the skin and immune cells but also the microbes present in the microbiota of the skin [1,2,3,4], which are crucial for proper wound healing; a sterile wound does not lead to a better healing outcome. Skin microbes play an important role in chemoattracting circulating neutrophils and monocytes to infiltrate the wounded tissue. Neutrophils release free oxygen species, cytotoxic enzymes, and proteases into the wound microenvironment and kill pathogens [5,6]. They are followed by monocytes that, when reaching the tissue, differentiate into pro-inflammatory macrophages which control microbial invasion and remove dead neutrophils and other cells. Subsequent to this phase of healing, pro-healing macrophages initiate the repair of the tissue by regulating inflammation, stimulating fibroblast proliferation, angiogenesis and keratinocytes’ proliferation and migration, to close the wound [7,8]. When chronic wounds develop, the tissue becomes ischemic, necrotic and hypoxic, providing a microenvironment that is conducive for the colonization and proliferation of potential pathogenic microbes. This also leads to chronic inflammation. It is in this destructive microenvironment that opportunistic pathogens take advantage of host nutrients and molecules to form a biofilm [9,10,11,12,13].

Bacterial biofilms can harbor a multitude of aerobic and anaerobic bacterial species from across the phylogenetic tree. Each species in the biofilm differs significantly from its planktonic counterpart in its morphology, mode of communication and metabolism [14,15,16]. The biofilm provides a unique environment for cell signaling through the production of quorum sensing molecules, which promote collective behavior such as the optimization of nutrients and acquisition and regulation of virulence, leading to sustained pathogenicity in the wound [12,17]. Further research on microbial biofilms is needed because the bacteria in these biofilms are becoming more resistant to conventional antibiotic therapies. They are also difficult to disassemble and deconstruct due to the complicated structure of the extracellular polymeric substances (EPSs) that comprise the biofilm matrix, which include extracellular DNA, proteins/peptides, carbohydrates and lipids [18,19,20].

Of the many bacterial species found to colonize chronic wounds in humans, *Pseudomonas aeruginosa* (*PA*) is a very common species that is difficult to control once it colonizes chronic wounds, because many strains of *PA* have already developed a resistance to antibiotics [21,22,23,24]. *PA* can form impenetrable biofilms that are difficult to remove even with the wide range of therapeutics available to clinicians. In addition, *PA* poses a significant health risk to patients because its genome contains a large arsenal of virulence factors that enhance pathogenesis [25,26,27]. Quorum sensing (QS) is an integral part of the pathogenesis of *PA* because it allows *PA* to adapt to diverse microenvironments. Two gene tandems are important for QS: *lasR/lasI* and *rhlR/rhlI* [28]. The transcription of these genes is highly expressed in their early stationary phase and their products can activate the expression of many other genes, including those involved in virulence.

*PA* can survive in both aerobic and anerobic conditions, but its most efficient growth occurs during aerobic respiration [29,30]. Due to this fast metabolism, the levels of its reactive oxygen species (ROS), e.g., superoxide (O_2_^·−^) and H_2_O_2_, which are dangerous by-products of aerobic respiration, must be controlled. *PA* has two superoxide dismutase (SOD) enzymes with either manganese or iron co-factors [29]. When iron levels are high, *sodB* expression, which encodes for the enzyme Fe-SOD, is high; otherwise, *sodA* expression, which encodes for the enzyme Mn-SOD, is activated. Both enzymes dismutate the highly damaging radical O_2_^·−^ to H_2_O_2_, which is less damaging. *PA* also expresses catalase, which is encoded by the two genes *katA* and *katB*, which breakdown H_2_O_2_ into H_2_O and O_2_ [29].

Chronic wounds in humans are a very serious condition; they have been considered by many as a silent epidemic that affects a large fraction of the world population [31]. Indeed, chronic wounds impact ~8.5 M people and cost ~USD 30 B/year in the US alone [29]. Many people with chronic wounds have other comorbidities, one of which is diabetes. Over 30 M people live with diabetes in the United States and it is projected that there will be 60.6 M diabetic Americans by 2060 [29]. Diabetic foot ulcers (DFUs) account for 25–50% of all diabetes-related hospital costs in the US, totaling billions of dollars per year [32]. *PA* is a large contributor to these wounds staying chronic because *PA* biofilms readily return after debridement. Therefore, it is important to understand how *PA* responds to the high oxidative stress environments in these wounds to form biofilms. Using both in vitro and in vivo approaches, we found that, in response to high levels of oxidative stress, *PA* expresses global transcriptional activators, antioxidant enzymes and SOD to break down ROS and expresses several genes that contribute to the formation of a biofilm. A bacterial transcriptomic analysis of chronic wounds showed that the Type VI Secretion System (T6SS) was significantly upregulated in chronic wounds. These results point to the potential of developing new approaches to target the *PA* genes contributing to its survival in vivo as a biofilm.

## 2. Materials and Methods

### 2.1. Pseudomonas aeruginosa’s Isolation, Growth and Treatment In Vitro

All experiments used Lysogeny Broth (LB; Difco, Franklin Lakes, NJ, USA) [33] as the growth medium. Aerobic liquid cultures were incubated at 37 °C and shaken at 220 RPM. For our studies, we used a *Pseudomonas aeruginosa* (*PA*) strain RPA (Riverside *PA*) that was previously isolated from the chronic wounds of our *db*/*db* mouse model of chronic wounds [34,35]. Briefly, the identity of RPA was confirmed after PCR amplification, cloning and Sanger sequencing. The in vitro experiments were conducted in sterile 96-well plates, with absorbance at OD_600_ used to measure the concentration of the bacteria colony forming units per ml (CFU/mL). Serial dilutions were made to achieve a concentration of 10^6^ CFU/200 µL and pipetted into each well. The 96-well plates were incubated at 37 °C without shaking. A bacterial treatment with hydrogen peroxide (H_2_O_2_) was performed by delivering 20 µL of a higher concentration of H_2_O_2_ to achieve a final concentration of 250 µM, 500 µM or 1000 µM of H_2_O_2_ in wells containing 200 µL of bacteria in LB. For the in vitro experiments, biofilm samples were not washed and contained both planktonic and attached cells. For in vivo experiments, absorbance at OD_600_ was used to measure the concentration of the bacteria to achieve a concentration of 10^7^ CFU/50 µL, which was applied on top of the wound 24 h after wounding.

### 2.2. Chronic Wounds Caused by Infection with RPA

All experiments were completed in accordance and compliance with federal regulations and the University of California’s policy and procedures. Animal experimental protocol no. 11 was approved in July 2023 by the University of California Riverside (UCR) Institutional Animal Care and Use Committee (UCR-IACUC). The description of how to obtain chronic wounds in *db*/*db* mice has been published in detail by us previously [35,36,37]. Briefly, *db*/*db* mice were bred in our conventional vivarium from B6.BKS(D)-*Lepr^db^*/J heterozygotes obtained from the Jackson Laboratories (Stock no. 000697). Only *db*/*db* that are at least 5 months old were used to create chronic wounds, because at this age they are fully diabetic and obese. Twenty min prior to performing the surgical wounds, 3-amino-1,2,4-triazole (ATZ), an inhibitor of catalase, was injected intraperitoneally at 0.75 g ATZ/kg of mouse weight in sterile PBS. The skin of the mouse was wiped and disinfected with iodine and 70% ethanol immediately prior to surgery to remove as much of the natural skin microbiota as possible. As soon as a 7 mm full thickness skin excision wound was made, it was covered with sterile Tegaderm, which provides a barrier to external contaminants and environmental bacteria from the cage and bedding. Using an insulin syringe, mercaptosuccinic acid (MSA), at 150 mg MSA/kg of mouse weight in sterile PBS, was deposited on top of the wound to inhibit glutathione peroxidase. All mice were treated for pain intraperitoneally with 0.05 mg buprenorphine/kg of mouse weight in sterile PBS before surgery and 6 h after surgery, and after that as needed.

### 2.3. RNA Collection from Mouse Wounds

RNA from the bacteria was collected from wounds at 24, 48, and 72 h after wounding, from both healing and chronic wounds. Briefly, 100 µL of sterile nuclease-free water was injected into the wound bed with an insulin syringe piercing through the Tegaderm. Immediately, 100 µL of exudate mixed with the sterile nuclease-free water was extracted with the same insulin syringe. Samples were stored in −80 °C before extraction with RNeasy Mini Kit Ref. 74106 (Qiagen, Venlo, The Netherlands).

### 2.4. RNA Extraction, DNase1 Treatment and RNA Cleanup

The total RNA extracted from the RPA strains was used for PCR and RT-qPCR amplification. RNA was extracted using the RNeasy Mini Kit Ref. 74106 (Qiagen) after the bacteria were sonicated for 30 sec in RLT buffer. The extracted total RNA was incubated for 3 h in DNaseI (Qiagen) and subsequently cleaned with vacuum grease and isopropyl alcohol. Briefly, the DNA-free RNA was added to a microcentrifuge tube with a vacuum grease phase lock. One volume of cold acid phenol was added, and the tube was hand-shaken to mix the water and acid phenol to obtain an emulsion. Samples were centrifuged for 4 min at 12,000 RPM. The aqueous phase was transferred to a new tube and combined with 1.5 µL of 15 mg/mL glycogen, 0.1X volume of 3 M sodium acetate, pH 5.2 and 1.2X volume of room-temperature isopropyl alcohol. The tube was mixed by inversion, not vortexed. Samples were incubated for 30 min at −20 °C or for 10 min at −80 °C before being centrifuged for 15 min at 12,000 RPM and 4 °C. The supernatant was removed while being careful not to disturb the white RNA pellet at the bottom of the tube. The pellet was rinsed with 500 µL of cold 75% ethanol and allowed to dry before dissolving the RNA with ultrapure nuclease-free water.

### 2.5. Reverse Transcription and Quantitative Polymerase Chain Reaction

The reverse transcriptase to obtain cDNA was conducted with the Superscript™ IV First-Strand Synthesis System (Invitrogen, Waltham, MA, USA). The primers used for the reaction are listed in Table 1. For RT-qPCR amplification, the total reaction volume was 20 µL, including 10 µL of SYBR Green (Biorad, Biotech, Dalian, China), 1 µL each of the forward and reverse primers (10 μM), 7 µL of sterile water, and 1 µL of the purified bacterial cDNA as a template. A Biorad CFX Connect System (Roche, Switzerland) was used for thermal cycling, as follows: an initial denaturation of DNA at 95 °C for 30 s, followed by 40 cycles of denaturation at 95 °C for 5 s and annealing at 55 °C for 60 s. The qPCR assay was performed in duplicates with parallel analysis, in 96-well plates. Sterile water was used in place of the DNA template as a negative control to ensure the absence of contaminants. Log_2_FC was calculated using *proC* as the stable housekeeping gene [38].

### 2.6. RNA Sequencing

The integrity of the DNase-treated RNA samples was assessed by a Bioanalyzer (Agilent, Hong Kong, China). All samples had RIN (RNA integrity number) values ≥ 5.0. Ribosomal transcripts were removed using the Illumina^®^ Stranded Total RNA Prep, Ligation with Ribo-Zero Plus (Illumina, cat no. 20040525, San Diego, CA, USA). Following the manufacturer’s instructions, 500 ng of total, DNase-treated RNA was used as the input for the ribo-depletion procedure. RNA samples were then barcoded with IDT^®^ for Illumina^®^ RNA UD Indexes Set A Ligation (96 Indexes, 96 Samples) (Illumina, cat no. 20040553). The quality of the barcoded libraries was assessed with a Bioanalyzer/Tapestation and pooled equilmolar before submission. Libraries were sequenced with Illumina NextSeq 2000, using P2 100-cycle kit (100 bp, Single End) for up to 4000 M reads with 20–25 M reads per sample.

### 2.7. Sequencing Genomic DNA and Annotations

Sequencing services were provided by SeqCoast Genomics, LLC (Portsmouth, NH, USA). The genomic DNA of RPA was sequenced first using the Illumina Sequencing Methods. Briefly, RPA sample libraries were prepared using the Illumina DNA Prep kit and IDT 10 bp UDI indices and sequenced on an Illumina NextSeq 2000, producing 2 × 151 bp reads. Demultiplexing, quality control and adapter trimming were performed with Illumina bcl-convert (v3.9.3). We then used the Oxford Nanopore Sequencing Method. Briefly, sample libraries were prepared using Oxford Nanopore Technologies (ONT) Ligation Sequencing Kit (SQK-LSK109), with NEBNext^®^ Companion Module (E7180L) in addition to Native Barcode Kits (EXP-NBD104, EXP-NBD114), to the manufacturer’s specifications. All samples were run on Nanopore R9.4.1 flow cells and a MinION Mk1B device. Post-sequencing, Guppy (v5.0.16) was used for high-accuracy base calling (HAC) and demultiplexing. Genome hybrid assembly was performed using Unicycler (version 0.4.4) [39], with error correction and contig assembly using SPAdes [40,41], mapping using Bowtie2 [42] and variant identification and assembly polishing with Pilot [43], which produced a single chromosomal contig of 6,839,924 bp and second plasmid contig of 7127 bp. Gene prediction and functional annotation were performed with BAKTA (version 1.5.1) [43,44,45,46,47,48,49,50,51,52,53,54,55,56,57,58].

### 2.8. Bioinformatic Analysis

To analyze the results, raw data were processed for quality control using Fastqc [59]. Low-quality bases and adapter sequences were removed using trim_galore (v0.6.7) [60]. High-quality trimmed reads were aligned to the RPA reference genome using the STAR aligner (v.2.7.9a) [61] with parameters –outFilterMismatchNmax 0 and –outFilterMultimapNmax 5 to select reads with a perfect match and that, at most, mapped to five locations. QC data were summarized using MultiQC (v1.16) [62]. Approximately 21–30 million reads were obtained per library, with the majority of the reads remaining after adapter trimming with Cutadapt. Fastq-screen (v0.15.2) [63] was used to sample each library (1 M reads) and quickly align them to known references including humans, mouse, *E. coli*, *Arabidopsis*, vectors, adapters, etc. This analysis provided a quick assessment of the extent of the contaminants in the libraries. Sortmerna (4.3.6) [64] was used to determine the extent of the rRNA contamination within each library. The reads were aligned against the rRNA references from the SILVA database. Featurecount (v2.0.3) [65] quantified the mapped reads with a genome annotation to generate a count matrix for each transcript/gene. The current featurecount setting only accepts uniquely mapped reads and ignores multiple-mapped reads. A differential expression analysis was performed using DESeq2 (1.38.0) [66]. Statistical significance was determined using log_2_FC > 1 and an adjusted *p*-value (FDR) < 0.1.

## 3. Results

### 3.1. Differential Gene Expression In Vitro between Biofilm-Forming PA and PA Already in a Biofilm

Biofilm-forming RPA was used to understand the response of *PA* to OS as it develops a biofilm in chronic wounds. RPA is a strain isolated from the biofilm of a fully chronic mouse wound that is very effective at forming a biofilm in vivo [34], and initial experiments showed that this strain also grows and produces a strong biofilm after 24 h under stressful conditions in vitro (incubation at 37 °C without shaking). To understand how RPA develops a biofilm, we first performed experiments in vitro in response to stress by investigating the transcriptional response of RPA to the OS created by H_2_O_2_ and compared the expression of genes known to be important for the response to stress and biofilm formation. Specifically, we compared the gene expression between planktonic bacteria as they develop into a biofilm (0–24 h) and their gene expression once they are already in a biofilm (24–48 h).

**Figure 1:**
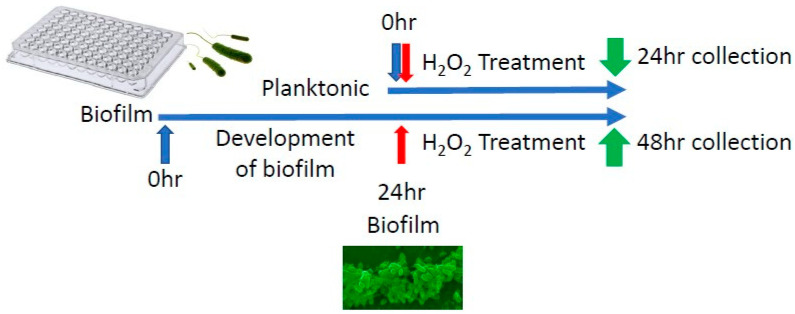
Experimental design of in vitro H_2_O_2_ treatment of RPA. RPA was grown in 96-well plates to test its transcriptomic response to H_2_O_2_ in a dose-dependent manner. Planktonic cells were treated for 24 h before they were collected for analysis. RPA, in biofilm form, was undisturbed for 24 h to allow it to form a biofilm. At 24 h it was treated with H_2_O_2_. After another 24 h of treatment, RPA cells in biofilm were collected for analysis.

**Figure 2:**
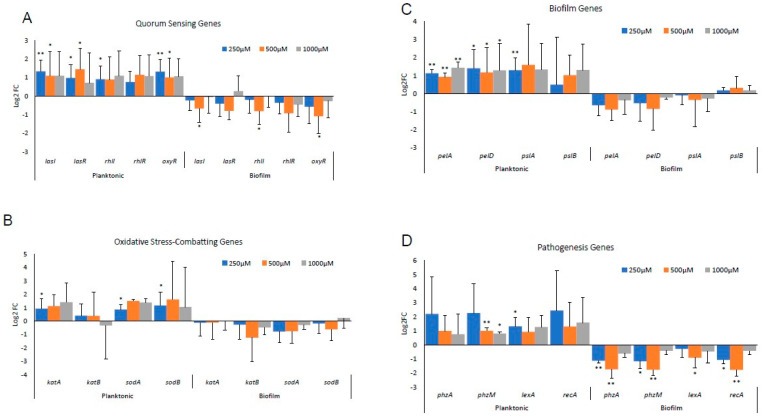
Transcriptional responses of planktonic RPA and of RPA in an in vitro biofilm culture when treated with H_2_O_2_. RPA was incubated with either H_2_O or 250 µM, 500 µM or 1000 µM of H_2_O_2_. Samples were collected in triplicate within 1 min for the 0 h timepoint and then at the specific times indicated in the figure. (**A**) Log_2_FC trends of important quorum sensing genes, *lasI* and *lasR*, and *rhlI* and *rhlR* and *oxyR,* that function in OS defense and DNA repair. (**B**) Log_2_FC trends of genes coding enzymes that metabolize H_2_O_2_: *katA* and *katB* and *sodA* and *sodB*. (**C**) Log_2_FC trends of critical biofilm genes for Pel formation, *pelA* and *pelD*, and Psl formation, *pslA* and *pslB*. (**D**) Log_2_FC trends of genes aiding in pathogenesis through the biosynthesis of a crucial redox-sensitive phenazine molecule, pyocyanin, *phzA* and *phzM,* and metabolism, *lexA* and *rpsL*. Bars represent average log_2_FC and error bars represent standard deviation. Statistical significance was calculated by comparing H_2_O_2_ treatments with the vehicle control using Student’s *t*-test. * *p*-value  <  0.05, ** *p*-value  <  0.01.

RPA was grown in sterile 96-well plates and treated with 250 µM, 500 µM or 1000 µM of H_2_O_2_ with a vehicle (sterile water) as control, to simulate bacterial exposure to low, medium, and high levels of OS. One set of cultures was treated from time zero of culture (planktonic) and the other allowed to form a biofilm for 24 h before treatments (Figure 1). Significant differences in gene expression were found between planktonic RPA cells growing to form a biofilm in response to OS and RPA already living in a biofilm culture when exposed to OS (Figure 2). In RPA planktonic cells, all QS genes, *lasI*, *lasR*, *rhlI* and *rhlR*, were upregulated (Figure 2A). The expression of *oxyR*, a gene that functions in OS defense and DNA repair, was also significantly upregulated. In contrast, these genes were downregulated when RPA was already in its biofilm form when exposed to H_2_O_2_ (Figure 2A). In particular, we observed that the expression of *lasI*, *lasR*, *rhlI*, *rhlR* and *oxyR,* when the bacteria were in a biofilm, was significantly downregulated with the 500 µM H_2_O_2_ treatment. The lack of the same strong downregulation when the bacteria were treated with 1000 µM could be due to these high levels of H_2_O_2_ causing toxicity to the bacteria.

*PA* has two SOD enzymes: *sodB,* which encodes for Fe-SOD, whose expression is high when iron is high; otherwise, *sodA*, which encodes for Mn-SOD, is highly expressed. Both enzymes dismutate the highly damaging radical O_2_^·−^ to H_2_O_2_, which is less damaging. *PA* also has two catalase genes that encode KatA and KatB, which breakdown H_2_O_2_ into H_2_O + O_2_ [29]. We found that, under planktonic conditions, *sodA*, *sodB* and *katA* are all highly upregulated, whereas, when in a biofilm, RPA does not activate these genes (Figure 2B).

When the genes responsible for Pel and Psl synthesis, *pelA* and *pelD* and *pslA* and *pslB*, respectively, were studied, we observed that, again, under planktonic conditions, these genes were upregulated, whereas, when the RPA was already in its biofilm, the same genes were downregulated, except for *pslB*, which was slightly upregulated (Figure 2C). Similar observations were made with the pathogenic genes, *phzA*, *phzB*, *lexA* and *rpsL* (Figure 2D).

### 3.2. Differential Gene Expression of Biofilm-Forming PA In Vivo

**Figure 3:**
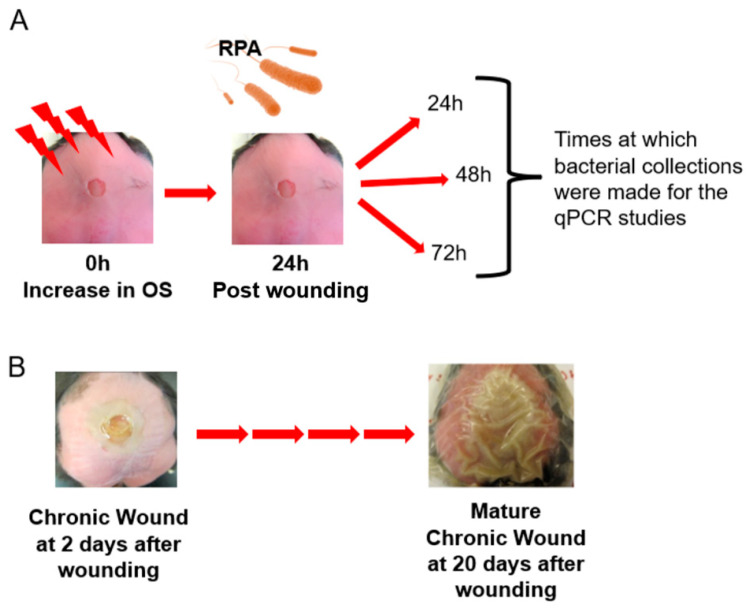
Chronic wound progression with RPA infection. RPA strain was isolated and identified from previous naturally infected chronic wounds. (**A**) For the in vivo studies, clean wounds were infected with RPA 24 h after surgery and the stimulation of chronicity. Bacterial samples were collected 24, 48 and 72 h after RPA was introduced into the wounds and analyzed for gene expression using qPCR. (**B**) Picture of a wound 48 h after injury which then progressed to a fully chronic wound.

**Figure 4:**
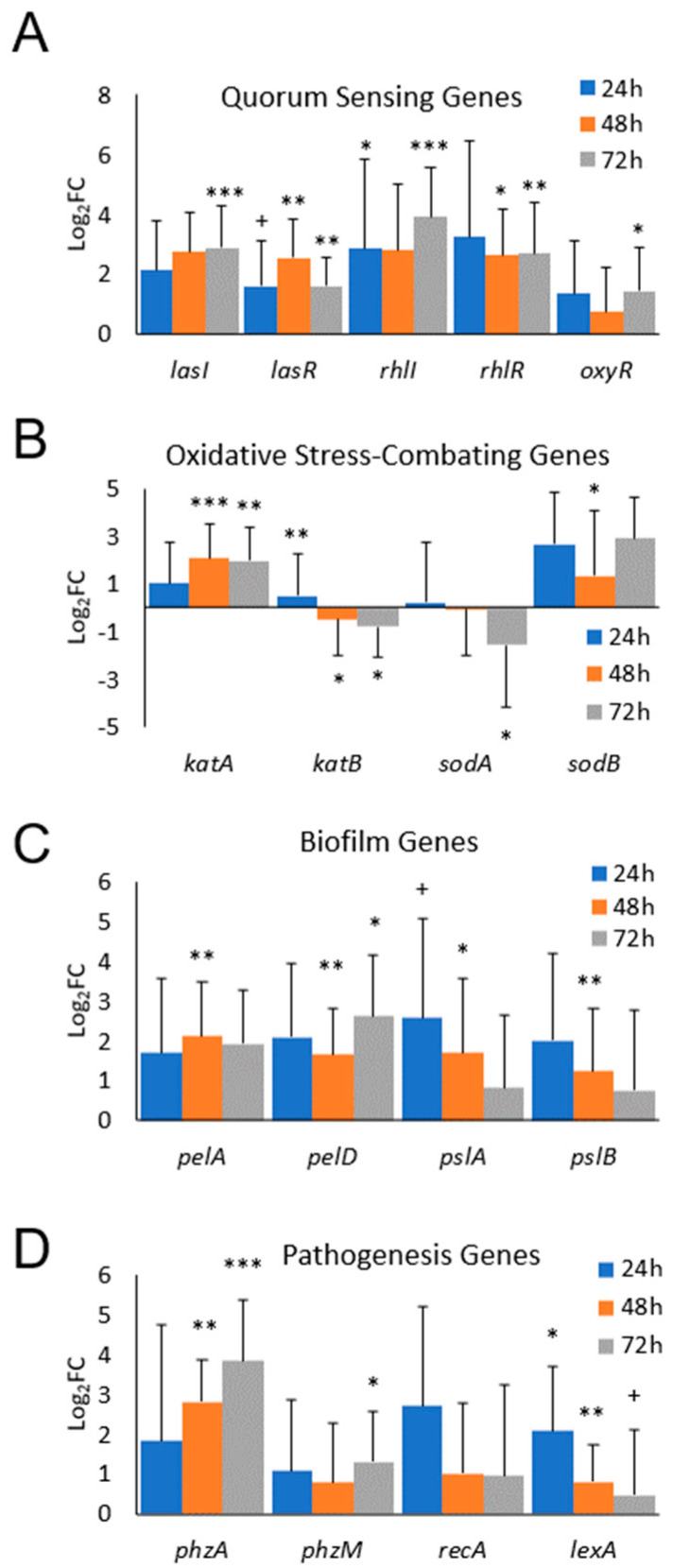
RPA activates quorum sensing, *OS-combating*, biofilm and pathogenesis genes in vivo. Non-chronic wounds (NCWs) and chronic wounds (CWs) were infected with RPA and the exudate was collected for RPA transcriptomics. Gene expression in CWs was compared to that in NCWs at 24 h, 48 h and 72 h (*n* = 6 at each timepoint for each condition, *n* = 36 total). (**A**) The log_2_FC of quorum sensing genes for *lasI*, *lasR*, *rhlI* and *rhlR* was significantly upregulated in CWs. *OxyR* was not significantly upregulated. (**B**) RPA preferentially expressed *katA* and *sodB* genes in CWs, whereas *katB* and *sodA* were significantly downregulated in CWs. (**C**) RPA upregulated the biofilm genes *pelA*, *pelD*, *pslA* and *pslB* in CWs. (**D**) *phzA* in the phenazine biosynthesis pathway was significantly expressed by RPA in CWs, whereas their *RecA* and *lexA* expression decreased over time. Bars represent average log_2_FC and error bars represent standard deviation. Statistical significance was calculated by comparing CWs with NCWs using Student’s *t*-test. * *p*-value  <  0.05, ** *p*-value  <  0.01, *** *p*-value < 0.001. + FDR < 0.1.

For the studies in vivo, we used our mouse model of chronic wounds, from which RPA was isolated [37]. Five-month-old, diabetic, obese *db*/*db* mice were used to perform excision wounds (Figure 3). For these experiments, the mouse skin was depleted of its microbiome, wounds were made and chronicity was induced. At 24 h after wounding, RPA was introduced under the Tegaderm and biofilm formation began to proceed in the wound environment. We observed an activation of all of the QS genes we tested, *lasI*, *lasR*, *rhlI*, *rhlR* and *oxyR,* by 24 h after the application of RPA (Figure 4A). However, for the antioxidant genes, we found that only *katA* and *sodB* were activated to decrease OS in the wound, whereas *katB* and *sodA* were downregulated (Figure 4B). The Pel and Psl genes that contribute to biofilm formation were all upregulated and so were all of the pathogenic genes we tested, *phzA*, *phzM*, *recA* and *lexA* (Figure 4C,D). *phzA* showed the highest upregulation, which increased with time as the wound progressed to chronicity.

### 3.3. Identification of Gene Expression in RPA-Induced Biofilm In Vivo by RNA-seq Detection

**Figure 5:**
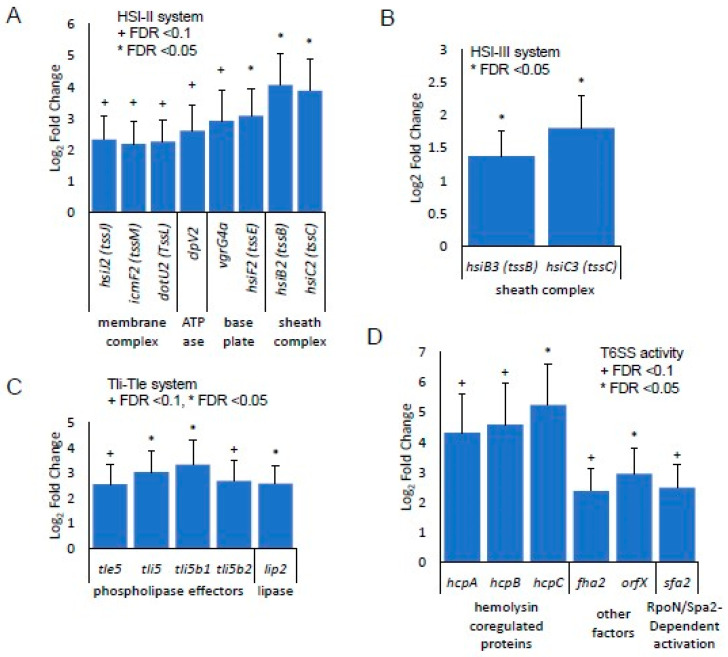
The Type VI Secretion System (T6SS) was significantly upregulated in chronic wounds. The RPA in chronic and nonchronic wound exudate was collected at 24 h, and its RNA was sequenced (*n* = 3 for both chronic wounds and nonchronic wounds, *n* = 6 total). (**A**) Many T6SS structural proteins of the HSI-II loci were found to be upregulated in chronic wounds, including those of the membrane complex (*hsiJ2*, *icmF2* and *dotU2*), ATPase (*clpV2*), the baseplate (*vgrG4a* and *hsiF2*) and the sheath complex (*hsiB2* and *hsiC2*). (**B**) Two components of the HIS-III sheath complex were significantly upregulated. (**C**) Effectors that have phospholipase activity, the Tli- Tle system, were also activated in CWs. (**D**) Hemolysin-coregulated proteins (*hcpA*, *hcpB* and *hcpC*) and other effectors (*fha2* and *orfX*) of T6SS were also upregulated. RpoN/Sfa2 may regulate T6SS activity. Bars represent average log_2_FC and error bars represent lfcSE. Statistical significance was determined using log_2_FC > 1 and an adjusted *p*-value (FDR) < 0.1, comparing CW to NCW samples. + FDR < 0.1, * FDR < 0.05.

**Figure 6:**
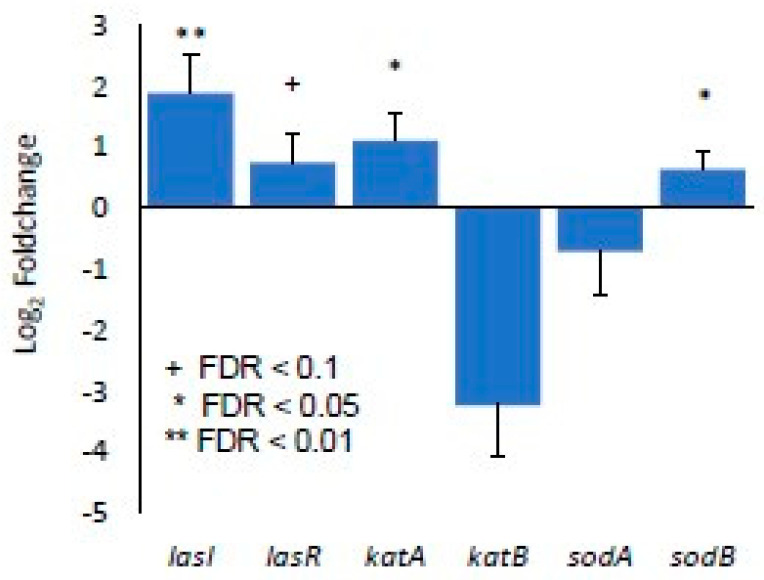
RNAseq confirmed gene expression: the differentially expressed quorum sensing genes, *lasI* and *lasR*, and antioxidant genes, *katA*, *katB*, *sodA* and *sodB*, obtained through RNAseq validate the genes expressed in vivo via the RT-qPCR results found in Figure 4. Bars represent average log_2_FC and error bars represent lfcSE. Statistical significance was determined using log_2_FC > 1 and an adjusted *p*-value (FDR) < 0.1, comparing CW to NCW samples. + FDR < 0.1, * FDR < 0.05, ** FDR < 0.01.

RPA-infected wounds were collected more than 24 h after inoculation for both chronic and nonchronic wounds at three timepoints: 24, 48 and 72 h after infection. Approximately 21–30 million reads were obtained per sample library and a FASTQC analysis of the raw reads from the sequenced libraries indicated that the reads are of high quality (e.g., few adapter sequences trimmed, overall base quality (q) > 30). The reads were aligned to the genome sequenced for RPA, with annotations predicted with BAKTA (see Materials and Methods). Over 150 genes were found to be significantly differentially expressed in the 24 h wound tissues, FDR < 0.1. No genes were observed to be differentially expressed at 48 h and 72 h and, therefore, these timepoints were excluded from subsequent analyses. Among the differentially expressed genes associated with metabolism and respiration at 24 h of infection, many genes of the Type VI Secretion System (T6SS) were significantly overexpressed (Figure 5). Most significantly, genes that participated in the biosynthesis of the secretion system’s HSI-II structure were upregulated in chronic wounds compared to nonchronic wounds (Figure 5A). Genes that form the membrane complex, *hsiJ2*, *icmF2* and *dotU2*; baseplate, *vgrG4a* and *hsiF2*; and sheath complex, *hsiB2* and *hsiC2*, were significantly upregulated. The ATPase that drives the secretion mechanism, *clpV2*, was also upregulated. In the sheath complex of HSI-III cluster, *hsiB3* and *hsiC3* were significantly upregulated (Figure 5B). Several genes responsible for the effectors of T6SS were significantly upregulated in chronic wounds at 24 h. Phospholipase effectors *tle5*, *tli5, tli5b1* and *tli5b2* were also significantly upregulated (Figure 5C). Several hemolysin coregulated proteins (hcp) and other effectors such as *fha2* and *orfX* were also upregulated in chronic wounds. RpoN/Sfa2-dependent activation may play a role in the regulation of the T6SS in the RPA in chronic wounds (Figure 5D). The quorum sensing genes *lasI* and *lasR* were upregulated in the RPA RNAseq analysis, confirming the results obtained through RT-qPCR. The preferential upregulation of *katA* and *sodB* seen in the RT-qPCR in vivo also correlated with the differentially expressed gene analysis after sequencing the RPA transcriptomics (Figure 6).

## 4. Discussion

*Pseudomonas aeruginosa* is a clinically significant, opportunistic pathogen in humans, and the strength of its pathogenesis in human infections relies on many virulence factors and effectors that are encoded in its large genome [67]. We have shown that RPA activates quorum sensing, virulence, biofilm formation and antioxidant enzyme genes in the high OS microenvironment present in chronic wounds and colonizes these wounds successfully by forming a biofilm. Our finding that RPA induces different genes when it exists as a biofilm serves as reminder that treatments in the clinic must appropriately target the metabolic state of biofilm-forming bacteria. Our transcriptomic analysis of the RPA in chronic wounds showed that many structural components and effectors of the recently described T6SS are upregulated. RPA could be using T6SS to destroy or modulate competitive bacteria, as well as to interact with and inactivate host immune cells and skin cells important for wound healing.

The importance of the different metabolic states of planktonic vs. biofilm RPA was reflected by their different responses to H_2_O_2_ in vitro. Planktonic RPA strongly activated its quorum sensing, OS response, virulence, biofilm formation and oxidative stress genes, whereas, in a mature biofilm, it did not upregulate any of these genes, suggesting that in biofilm conditions *PA* is already in a physiologic state that is not affected by the inhospitable environment of chronic wounds. Therefore, it is possible that the activation of these genes could put the bacteria in a “planktonic” state and render them more susceptible to standard antibiotic treatments.

The in vivo model of *PA* infection showed that RPA initially survives the harsh, high OS microenvironment present in chronic wounds and colonizes these wounds as a biofilm by turning on the quorum sensing genes (Figure 5A) responsible for coordinating the genes that contribute to biofilm development (Figure 5C). *pelD*, for example, is an essential gene for Pel polysaccharide production. It regulates the production of the polysaccharide through c-di-GMP, a second messenger used in signal transduction that controls the cellular processes that contribute to surface adaptation, biofilm formation, cell cycle progression and virulence [68]. *pelA* is an important gene necessary for the modification of the Pel polymer after its assembly and secretion through a complex modified after polymerization in the periplasm [68]. Note that both *pelA* and *pelD* are upregulated by an exposure to H_2_O_2_ in vitro (Figure 2D).

Four antioxidant enzymes critical for the removal or breakdown of hydrogen peroxide were found to be differentially expressed both in vitro and in vivo. The gene expression of two SOD genes, *sodA* (manganese cofactor) and *sodB* (iron cofactor), showed that sodB may be more important than sodA for aerobic growth [68]. *SodB* expression was upregulated, while *sodA* expression was downregulated. This could be explained by the fact that, in the early stages of the response to injury, iron is very abundant in the wound [69,70] and can be used by SodB for its activation [71,72,73]. In addition, the controlled regulation of the lasR/lasI and rhlR/rhlI system has been found to be crucial for *PA* to form a biofilm and respond to oxidative stress [68]. A mutation in either or both of these systems results in *PA* becoming more sensitive to H_2_O_2_ because the expression levels of the genes for *sodA*, *sodB* and *katA* are decreased [74]. For the catalase genes required for a resistance to peroxides and osmotic stresses, we found that *katA* was significantly upregulated with exposure to the higher concentration of hydrogen peroxide, whereas *katB* expression was significantly downregulated. This is not surprising, because katA is the major catalase of *PA* that detoxifies H_2_O_2_, a reactive oxygen species that is generated during aerobic respiration [75]. 

In addition to QS, SOD and catalase, which maximize growth under excess OS conditions, *PA* also produces enzymes such as alkaline protease, elastase and lasA protease, and metabolites such hemolysin, rhamnolipids and phenazines (e.g., pyocyanin (PYO)), to combat host immune cells during infection [29,76]. PYO is a blue secondary metabolite; its synthesis is partly regulated by QS. As a zwitterion, it can easily penetrate and cross biological membranes. It is a powerful metabolite because it is redox-active and known to play a crucial role in the virulence and infection of *PA* in humans and in animal models. PYO can inactivate catalase, but not SOD, in a human epithelial cell line [77]. It modulates the normal glutathione (GSH) cycle by depleting cellular GSH and increasing oxidized GSH (GSSG) in epithelial cells [78]. The synthesis of PYO is controlled by two phenazine operons (*phzABCDEFG*) and by the *phzH*, *phzM* and *phzS* genes [79]. In addition, two other systems are involved in *PA*’s virulence and biofilm formation: the Pel and Psl systems of exopolysaccharides, which help facilitate the formation of biofilms while impairing bacterial clearance [28].

Our bacterial transcriptomic analysis of RPA during the first 24 h of chronic wound initiation showed that the T6SS was significantly upregulated. This system is a recently described system of *PA* which adds another virulent weapon to its ever-growing arsenal to compete and thrive in diverse environments. The T6SS has several structural components including a puncturing structure that has been described in bacteriophages [80]. *PA* benefits from its T6SS by delivering toxins to its neighboring pathogens and translocating protein effectors into the host cells. The T6SS also takes part in biofilm formation [80]. *PA* has been found to use the T6SS to compete in multi-bacterial species communities, providing growing and fitness advantages over other species in a mixed-species biofilm [81]. *PA* has been described to have the ability to invade and infect eukaryotic cells using this secretion system. Mutations in the system have shown a decreased or weakened virulence phenotype in a rat model of chronic lung infection [80,82]. *PA* also has the ability to target and infect human lung epithelial cells through quorum sensing Las and Rhl systems and induces the host Akt pathway, mediated through the phosphatidylinositol 3-kinase-dependent pathway [83].

**In conclusion**, *Pseudomonas aeruginosa* activates quorum sensing, OS-combating, biofilm-forming and virulence genes in the high OS microenvironment present in chronic wounds and colonizes these wounds successfully by forming a biofilm. The T6SS provides an opportunity to target the ability of *PA* uses to destroy other bacteria in the biofilm as well as host cells. Effective biofilm removal may be accomplished by disrupting these systems and dismantling the biofilm, leaving the planktonic bacteria unprotected and susceptible to antibiotic treatment.

## Figures and Tables

**Table 1:** List of Primers used for RT-qPCR.

Gene	Forward Primer	Reverse Primer	Gene Accession Number
*sodA*	CAACCACTCGCTGTTCTGGA	CTTGGTGAACGCATCCTTGAAC	PA4468
*sodB*	AACACCTACGTGGTGAACCTGA	TGACGATCTCTTCGAGGCTCTT	PA4366
*katA*	GAACAGCTTCAACCAGTGGCAG	CTCGTCGGTGAACAGATGGAAC	PA4236
*katB*	CGCTTCGATTTCTTCTCCCACG	CTTGTAGGCATGCACGCTGTTG	PA4613
*lasI*	GCCCCTACATGCTGAAGAACAC	CCTCCAGCGTACAGTCGGAAAA	PA1432
*lasR*	ATGGCCTTGGTTGACGGTTTTC	CCTAAGGACAGCCAGGACTACG	PA1430
*rhlR*	CCTCGGAAATGGTGGTCTGGAG	GGAAAGCACGCTGAGCAAATTG	PA3477
*rhlI*	CGACCAGGAATTCGACCAGTTC	GTTTCGCTGCACAGGTAGGC	PA3476
*pelA*	AGTACTACGCGCCGATCATCAA	AAGTGGTAGTACAGGTGCAGGC	PA3064
*pelD*	TGCCTGTATGCCTTCGAGTTGA	GAAGTCAGCGGCAACAACACC	PA3061
*pslA*	GCTACAACAACCGGCTGATCTG	GATGCTGGTCTTGCGGATGAAG	PA2231
*pslB*	CCTCAACACCAACGAATCCACC	CGTAGATGTCGTTGAAGCGGAC	PA2232
*recA*	TCACCGGCAATATCAAGAACGC	GACCGAGGCGTAGAACTTCAGT	PA3617
*lexA*	GCGAGGAGGTCACGGTGAAA	GCCTTCGATGATCAGTTCCTGC	PA3007
*proC*	CAGGCCGGGCAGTTGCTGTC	GGTCAGGCGCGAGGCTGTCT	PA0393
*oxyR*	CCGCTGTACATCGAGGAGAACT	ACATAGAAGGGCTCGTCGAACA	PA5344
*phzA1&2*	GACCGAGGATCCGAACCACTTC	CGTTTTATCCGGCCGTTCTCG	PA4210, PA1899
*phzM*	GTGGCCTTCGAGATCTTCCAGG	GGAACTCCTCGCCGTAGAACAG	PA4209

## Data Availability

The bacterial genome sequences for RPA have been deposited in the National Center for Biotechnology Information (NCBI)’s Sequence Read Archive (SRA) under the BioProject Accession Number PRJNA1112363. The bacterial RNAseq sequences and raw count matrix file have been deposited in NCBI’s Gene Expression Omnibus (GEO) under the GEO Accession Number GSE267862.

## References

[1] Kim J.H.H., Ahamed A., Chen K., Lebig E.G.G., Petros B., Saeed S., Martins-Green M. (2022). Skin microbiota and its role in health and disease with an emphasis on wound healing and chronic wound development. Microbiome, Immunity, Digestive Health and Nutrition: Epidemiology, Pathophysiology, Prevention and Treatment.

[2] Loomis K.H., Wu S.K., Ernlund A., Zudock K., Reno A., Blount K., Karig D.K. (2021). A mixed community of skin microbiome representatives influences cutaneous processes more than individual members. Microbiome.

[3] Khanna S., Biswas S., Shang Y., Collard E., Azad A., Kauh C., Bhasker V., Gordillo G.M., Sen C.K., Roy S. (2010). Macrophage dysfunction impairs resolution of inflammation in the wounds of diabetic mice. PLoS ONE.

[4] Flowers L., Grice E.A. (2020). The Skin Microbiota: Balancing Risk and Reward. Cell Host Microbe.

[5] Egozi E.I., Ferreira A.M., Burns A.L., Gamelli R.L., Dipietro L.A. (2003). Mast cells modulate the inflammatory but not the proliferative response in healing wounds. Wound Repair Regen..

[6] Wlaschek M., Peus D., Achterberg V., Meyer-Ingold W., Scharffetter-Kochanek K. (1997). Protease inhibitors protect growth factor activity in chronic wounds. Br. J. Dermatol..

[7] Kim S.Y., Nair M.G. (2019). Macrophages in wound healing: Activation and plasticity. Immunol. Cell Biol..

[8] Ridiandries A., Tan J.T.M., Bursill C.A. (2018). The role of chemokines in wound healing. Int. J. Mol. Sci..

[9] Gjødsbøl K., Christensen J.J., Karlsmark T., Jørgensen B., Klein B.M., Krogfelt K.A. (2006). Multiple bacterial species reside in chronic wounds: A longitudinal study. Int. Wound J..

[10] James G.A., Swogger E., Wolcott R., Pulcini E.D., Secor P., Sestrich J., Costerton J.W., Stewart P.S. (2008). Biofilms in chronic wounds. Wound Repair Regen..

[11] Schäfer M.M., Werner S., SCHAFER M., Werner S. (2008). Oxidative stress in normal and impaired wound repair. Pharmacol. Res..

[12] Zhao G., Usui M.L., Lippman S.I., James G.A., Stewart P.S., Fleckman P., Olerud J.E., Zhao G., Lippman S.I., James G.A. (2013). Biofilms and Inflammation in Chronic Wounds. Adv. Wound Care.

[13] Wolcott R.D., Rhoads D.D., Dowd S.E. (2008). Biofilms and chronic wound inflammation. J. Wound Care.

[14] Burmølle M., Thomsen T.R., Fazli M., Dige I., Christensen L., Homøe P., Tvede M., Nyvad B., Tolker-Nielsen T., Givskov M. (2010). Biofilms in chronic infections—A matter of opportunity–monospecies biofilms in multispecies infections. FEMS Immunol. Med. Microbiol..

[15] Leaper D., Assadian O., Edmiston C.E. (2015). Approach to chronic wound infections. Br. J. Dermatol..

[16] Phalak P., Chen J., Carlson R.P., Henson M.A. (2016). Metabolic modeling of a chronic wound biofilm consortium predicts spatial partitioning of bacterial species. BMC Syst. Biol..

[17] Stewart P.S.S., Franklin M.J.J. (2008). Physiological heterogeneity in biofilms. Nat. Rev. Microbiol..

[18] Brandwein M., Steinberg D., Meshner S. (2016). Microbial biofilms and the human skin microbiome. npj Biofilms Microbiomes.

[19] Sutherland I.W. (2001). Biofilm exopolysaccharides: A strong and sticky framework. Microbiology.

[20] Billings N., Ramirez Millan M., Caldara M., Rusconi R., Tarasova Y., Stocker R., Ribbeck K. (2013). The Extracellular Matrix Component Psl Provides Fast-Acting Antibiotic Defense in *Pseudomonas aeruginosa* Biofilms. PLoS Pathog..

[21] Zhao G., Hochwalt P.C., Usui M.L., Underwood R.A., Singh P.K., James G.A., Stewart P.S., Fleckman P., Olerud J.E. (2010). Delayed wound healing in diabetic (db/db) mice with *Pseudomonas aeruginosa* biofilm challenge: A model for the study of chronic wounds. Wound Repair Regen..

[22] Nagoba B.S., Suryawanshi N.M., Wadher B., Selkar S. (2015). Acidic Environment and Wound Healing: A Review. Wounds.

[23] Harrington N.E., Sweeney E., Harrison F. (2020). Building a better biofilm–Formation of in vivo-like biofilm structures by *Pseudomonas aeruginosa* in a porcine model of cystic fibrosis lung infection. Biofilm.

[24] Withycombe C., Purdy K.J., Maddocks S.E. (2017). Micro-management: Curbing chronic wound infection. Mol. Oral Microbiol..

[25] El-Khashaab T.H., Erfan D.M., Kamal A. (2016). *Pseudomonas aeruginosa* Biofilm Formation and Quorum Sensing lasR Gene in Patients with Wound Infection. Egypt. J. Med. Microbiol..

[26] Olejnickova K., Hola V., Ruzicka F. (2010). Virulence factors of *Pseudomonas aeruginosa* strains isolated from catheterized patients. Clin. Microbiol. Infect..

[27] Caldwell C.C., Chen Y., Goetzmann H.S., Hao Y., Borchers M.T., Hassett D.J., Young L.R., Mavrodi D., Thomashow L., Lau G.W. (2009). *Pseudomonas aeruginosa* exotoxin pyocyanin causes cystic fibrosis airway pathogenesis. Am. J. Pathol..

[28] Stedfeld R. (1978). How To Build a Better Bus. Mater. Eng..

[29] Arai H. (2011). Regulation and Function of Versatile Aerobic and Anaerobic Respiratory Metabolism in *Pseudomonas aeruginosa*. Front. Microbiol..

[30] Spero M.A., Newman D.K. (2018). Chlorate Specifically Targets Oxidant-Starved, Antibiotic-Tolerant Populations of *Pseudomonas aeruginosa* Biofilms. mBio.

[31] Sen C.K. (2021). Human Wound and Its Burden: Updated 2020 Compendium of Estimates. Adv. Wound Care.

[32] Hicks C.W., Selvarajah S., Mathioudakis N., Sherman R.L., Hines K.F., Black J.H., Abularrage C.J. (2016). Burden of Infected Diabetic Foot Ulcers on Hospital Admissions and Costs. Ann. Vasc. Surg..

[33] MacWilliams M.P., Liao M. (2006). Luria Broth (LB) and Luria Agar (LA) Media and Their Uses Protocol.

[34] Kim J.H., Spero M., Lebig E.G., Lonergan Z.R., Trindade I.B., Newman D.K., Martins-Green M. (2023). Targeting Anaerobic Respiration in *Pseudomonas aeruginosa* with Chlorate Improves Healing of Chronic Wounds. Adv. Wound Care.

[35] Kim J.H., Ruegger P.R., Lebig E.G., VanSchalkwyk S., Jeske D.R., Hsiao A., Borneman J., Martins-Green M. (2020). High Levels of Oxidative Stress Create a Microenvironment That Significantly Decreases the Diversity of the Microbiota in Diabetic Chronic Wounds and Promotes Biofilm Formation. Front. Cell. Infect. Microbiol..

[36] Kim J.H., Martins-Green M. (2019). Protocol to Create Chronic Wounds in Diabetic Mice. J. Vis. Exp..

[37] Dhall S., Do D.C., Garcia M., Kim J., Mirebrahim S.H., Lyubovitsky J., Lonardi S., Nothnagel E.A., Schiller N., Martins-Green M. (2014). Generating and reversing chronic wounds in diabetic mice by manipulating wound redox parameters. J. Diabetes Res..

[38] Savli H., Karadenizli A., Kolayli F., Gundes S., Ozbek U., Vahaboglu H. (2003). Expression stability of six housekeeping genes: A proposal for resistance gene quantification studies of *Pseudomonas aeruginosa* by real-time quantitative RT-PCR. J. Med. Microbiol..

[39] Wick R.R., Judd L.M., Gorrie C.L., Holt K.E. (2017). Unicycler: Resolving bacterial genome assemblies from short and long sequencing reads. PLoS Comput. Biol..

[40] Bankevich A., Nurk S., Antipov D., Gurevich A.A., Dvorkin M., Kulikov A.S., Lesin V.M., Nikolenko S.I., Pham S., Prjibelski A.D. (2012). SPAdes: A New Genome Assembly Algorithm and Its Applications to Single-Cell Sequencing. J. Comput. Biol..

[41] Nurk S., Meleshko D., Korobeynikov A., Pevzner P.A. (2017). metaSPAdes: A new versatile metagenomic assembler. Genome Res..

[42] Langmead B., Salzberg S.L. (2012). Fast gapped-read alignment with Bowtie 2. Nat. Methods.

[43] Walker B.J., Abeel T., Shea T., Priest M., Abouelliel A., Sakthikumar S., Cuomo C.A., Zeng Q., Wortman J., Young S.K. (2014). Pilon: An Integrated Tool for Comprehensive Microbial Variant Detection and Genome Assembly Improvement. PLoS ONE.

[44] Schwengers O., Jelonek L., Dieckmann M.A., Beyvers S., Blom J., Goesmann A. (2021). Bakta: Rapid and standardized annotation of bacterial genomes via alignment-free sequence identification. Microb. Genom..

[45] Seemann T. (2014). Prokka: Rapid prokaryotic genome annotation. Bioinformatics.

[46] Hyatt D., Chen G.-L., LoCascio P.F., Land M.L., Larimer F.W., Hauser L.J. (2010). Prodigal: Prokaryotic gene recognition and translation initiation site identification. BMC Bioinform..

[47] Siguier P. (2006). ISfinder: The reference centre for bacterial insertion sequences. Nucleic Acids Res..

[48] Chan P.P., Lowe T.M. (2019). tRNAscan-SE: Searching for tRNA Genes in Genomic Sequences. Gene Prediction: Methods and Protocols.

[49] Edgar R.C. (2007). PILER-CR: Fast and accurate identification of CRISPR repeats. BMC Bioinform..

[50] Feldgarden M., Brover V., Gonzalez-Escalona N., Frye J.G., Haendiges J., Haft D.H., Hoffmann M., Pettengill J.B., Prasad A.B., Tillman G.E. (2021). AMRFinderPlus and the Reference Gene Catalog facilitate examination of the genomic links among antimicrobial resistance, stress response, and virulence. Sci. Rep..

[51] Savojardo C., Martelli P.L., Fariselli P., Casadio R. (2018). DeepSig: Deep learning improves signal peptide detection in proteins. Bioinformatics.

[52] Laslett D. (2004). ARAGORN, a program to detect tRNA genes and tmRNA genes in nucleotide sequences. Nucleic Acids Res..

[53] Nawrocki E.P., Eddy S.R. (2013). Infernal 1.1: 100-fold faster RNA homology searches. Bioinformatics.

[54] Yang Z. (2007). PAML 4: Phylogenetic Analysis by Maximum Likelihood. Mol. Biol. Evol..

[55] Suyama M., Torrents D., Bork P. (2006). PAL2NAL: Robust conversion of protein sequence alignments into the corresponding codon alignments. Nucleic Acids Res..

[56] Miller S.R., Abresch H.E., Ulrich N.J., Sano E.B., Demaree A.H., Oman A.R., Garber A.I. (2021). Bacterial adaptation by a transposition burst of an invading IS element. Genome Biol. Evol..

[57] Garber A. (2021). BagOfTricks. GitHub Repos. https://github.com/Arkadiy-Garber/BagOfTricks.

[58] Krzywinski M., Schein J., Birol İ., Connors J., Gascoyne R., Horsman D., Jones S.J., Marra M.A. (2009). Circos: An information aesthetic for comparative genomics. Genome Res..

[59] Andrews S. (2010). FastQC: A Quality Control Tool for High Throughput Sequence Data.

[60] Krueger F. (2015). Trim Galore. A Wrapper Tool Around Cutadapt and FastQC to Consistently Apply Quality and Adapter Trimming to FastQ Files.

[61] Dobin A., Davis C.A., Schlesinger F., Drenkow J., Zaleski C., Jha S., Batut P., Chaisson M., Gingeras T.R. (2013). STAR: Ultrafast universal RNA-seq aligner. Bioinformatics.

[62] Ewels P., Magnusson M., Lundin S., Käller M. (2016). MultiQC: Summarize analysis results for multiple tools and samples in a single report. Bioinformatics.

[63] Wingett S.W., Andrews S. (2018). FastQ Screen: A tool for multi-genome mapping and quality control. F1000Research.

[64] Kopylova E., Noé L., Touzet H. (2012). SortMeRNA: Fast and accurate filtering of ribosomal RNAs in metatranscriptomic data. Bioinformatics.

[65] Liao Y., Smyth G.K., Shi W. (2014). featureCounts: An efficient general purpose program for assigning sequence reads to genomic features. Bioinformatics.

[66] Love M.I., Huber W., Anders S. (2014). Moderated estimation of fold change and dispersion for RNA-seq data with DESeq2. Genome Biol..

[67] Stover C.K., Pham X.Q., Erwin A.L., Mizoguchi S.D., Warrener P., Hickey M.J., Brinkman F.S.L., Hufnagle W.O., Kowalik D.J., Lagrou M. (2000). Complete genome sequence of *Pseudomonas aeruginosa* PAO1, an opportunistic pathogen. Nature.

[68] Whitfield G.B., Marmont L.S., Ostaszewski A., Rich J.D., Whitney J.C., Parsek M.R., Harrison J.J., Howell P.L. (2020). Pel Polysaccharide Biosynthesis Requires an Inner Membrane Complex Comprised of PelD, PelE, PelF, and PelG. J. Bacteriol..

[69] Yeoh-Ellerton S., Stacey M.C. (2003). Iron and 8-Isoprostane Levels in Acute and Chronic Wounds. J. Investig. Dermatol..

[70] Wright J.A., Richards T., Srai S.K.S. (2014). The role of iron in the skin and cutaneous wound healing. Front. Pharmacol..

[71] Wilderman P.J., Sowa N.A., FitzGerald D.J., FitzGerald P.C., Gottesman S., Ochsner U.A., Vasil M.L. (2004). Identification of tandem duplicate regulatory small RNAs in *Pseudomonas aeruginosa* involved in iron homeostasis. Proc. Natl. Acad. Sci. USA.

[72] Cavinato L., Genise E., Luly F.R., Di Domenico E.G., Del Porto P., Ascenzioni F. (2020). Escaping the Phagocytic Oxidative Burst: The Role of SODB in the Survival of *Pseudomonas aeruginosa* Within Macrophages. Front. Microbiol..

[73] da Cruz Nizer W.S., Inkovskiy V., Versey Z., Strempel N., Cassol E., Overhage J. (2021). Oxidative Stress Response in *Pseudomonas aeruginosa*. Pathogens.

[74] Hassett D.J., Ma J.-F., Elkins J.G., McDermott T.R., Ochsner U.A., West S.E.H., Huang C.-T., Fredericks J., Burnett S., Stewart P.S. (1999). Quorum sensing in *Pseudomonas aeruginosa* controls expression of catalase and superoxide dismutase genes and mediates biofilm susceptibility to hydrogen peroxide. Mol. Microbiol..

[75] Su S., Panmanee W., Wilson J.J., Mahtani H.K., Li Q., VanderWielen B.D., Makris T.M., Rogers M., McDaniel C., Lipscomb J.D. (2014). Catalase (KatA) Plays a Role in Protection against Anaerobic Nitric Oxide in *Pseudomonas aeruginosa*. PLoS ONE.

[76] Lau G.W., Hassett D.J., Ran H., Kong F. (2004). The role of pyocyanin in *Pseudomonas aeruginosa* infection. Trends Mol. Med..

[77] O’Malley Y.Q., Reszka K.J., Rasmussen G.T., Abdalla M.Y., Denning G.M., Britigan B.E. (2003). The Pseudomonas secretory product pyocyanin inhibits catalase activity in human lung epithelial cells. Am. J. Physiol. Cell. Mol. Physiol..

[78] O’Malley Y.Q., Reszka K.J., Spitz D.R., Denning G.M., Britigan B.E. (2004). *Pseudomonas aeruginosa* pyocyanin directly oxidizes glutathione and decreases its levels in airway epithelial cells. Am. J. Physiol. Cell. Mol. Physiol..

[79] Mavrodi D.V., Bonsall R.F., Delaney S.M., Soule M.J., Phillips G., Thomashow L.S. (2001). Functional analysis of genes for biosynthesis of pyocyanin and phenazine-1-carboxamide from *Pseudomonas aeruginosa* PAO1. J. Bacteriol..

[80] Filloux A., Hachani A., Bleves S. (2008). The bacterial type VI secretion machine: Yet another player for protein transport across membranes. Microbiology.

[81] Cheng Y., Yam J.K.H., Cai Z., Ding Y., Zhang L.-H., Deng Y., Yang L. (2019). Population dynamics and transcriptomic responses of *Pseudomonas aeruginosa* in a complex laboratory microbial community. npj Biofilms Microbiomes.

[82] Potvin E., Lehoux D.E., Kukavica-Ibrulj I., Richard K.L., Sanschagrin F., Lau G.W., Levesque R.C. (2003). In vivo functional genomics of *Pseudomonas aeruginosa* for high-throughput screening of new virulence factors and antibacterial targets. Environ. Microbiol..

[83] Sana T.G., Hachani A., Bucior I., Soscia C., Garvis S., Termine E., Engel J., Filloux A., Bleves S. (2012). The second type VI secretion system of *Pseudomonas aeruginosa* strain PAO1 is regulated by quorum sensing and fur and modulates internalization in epithelial cells. J. Biol. Chem..

